# Genomic prediction of yield and root development in wheat under changing water availability

**DOI:** 10.1186/s13007-020-00634-0

**Published:** 2020-07-01

**Authors:** Xiangyu Guo, Simon F. Svane, Winnie S. Füchtbauer, Jeppe R. Andersen, Just Jensen, Kristian Thorup-Kristensen

**Affiliations:** 1 Center for Quantitative Genetics and Genomics, Aarhus University, 8830 Tjele, Denmark; 2 Department of Plant and Environmental Science, University of Copenhagen, 1871 Frederiksberg, Denmark; 3 Sejet Plant Breeding I/S, 8700 Horsens, Denmark; 4 Nordic Seed A/S, 8300 Odder, Denmark

**Keywords:** Genomic prediction, Wheat, Semi-field, Grain-related yield, Deep root

## Abstract

**Background:**

Deeper roots help plants take up available resources in deep soil ensuring better growth and higher yields under conditions of drought. A large-scale semi-field root phenotyping facility was developed to allow a water availability gradient and detect potential interaction of genotype by water availability gradient. Genotyped winter wheat lines were grown as rows in four beds of this facility, where indirect genetic effects from neighbors could be important to trait variation. The objective was to explore the possibility of genomic prediction for grain-related traits and deep root traits collected via images taken in a minirhizotron tube under each row of winter wheat measured.

**Results:**

The analysis comprised four grain-related traits: grain yield, thousand-kernel weight, protein concentration, and total nitrogen content measured on each half row that were harvested separately. Two root traits, total root length between 1.2 and 2 m depth and root length in four intervals on each tube were also analyzed. Two sets of models with or without the effects of neighbors from both sides of each row were applied. No interaction between genotypes and changing water availability were detected for any trait. Estimated genomic heritabilities ranged from 0.263 to 0.680 for grain-related traits and from 0.030 to 0.055 for root traits. The coefficients of genetic variation were similar for grain-related and root traits. The prediction accuracy of breeding values ranged from 0.440 to 0.598 for grain-related traits and from 0.264 to 0.334 for root traits. Including neighbor effects in the model generally increased the estimated genomic heritabilities and accuracy of predicted breeding values for grain yield and nitrogen content.

**Conclusions:**

Similar relative amounts of additive genetic variance were found for both yield traits and root traits but no interaction between genotypes and water availability were detected. It is possible to obtain accurate genomic prediction of breeding values for grain-related traits and reasonably accurate predicted breeding values for deep root traits using records from the semi-field facility. Including neighbor effects increased the estimated additive genetic variance of grain-related traits and accuracy of predicting breeding values. High prediction accuracy can be obtained although heritability is low.

## Background

**Fig. 1:**
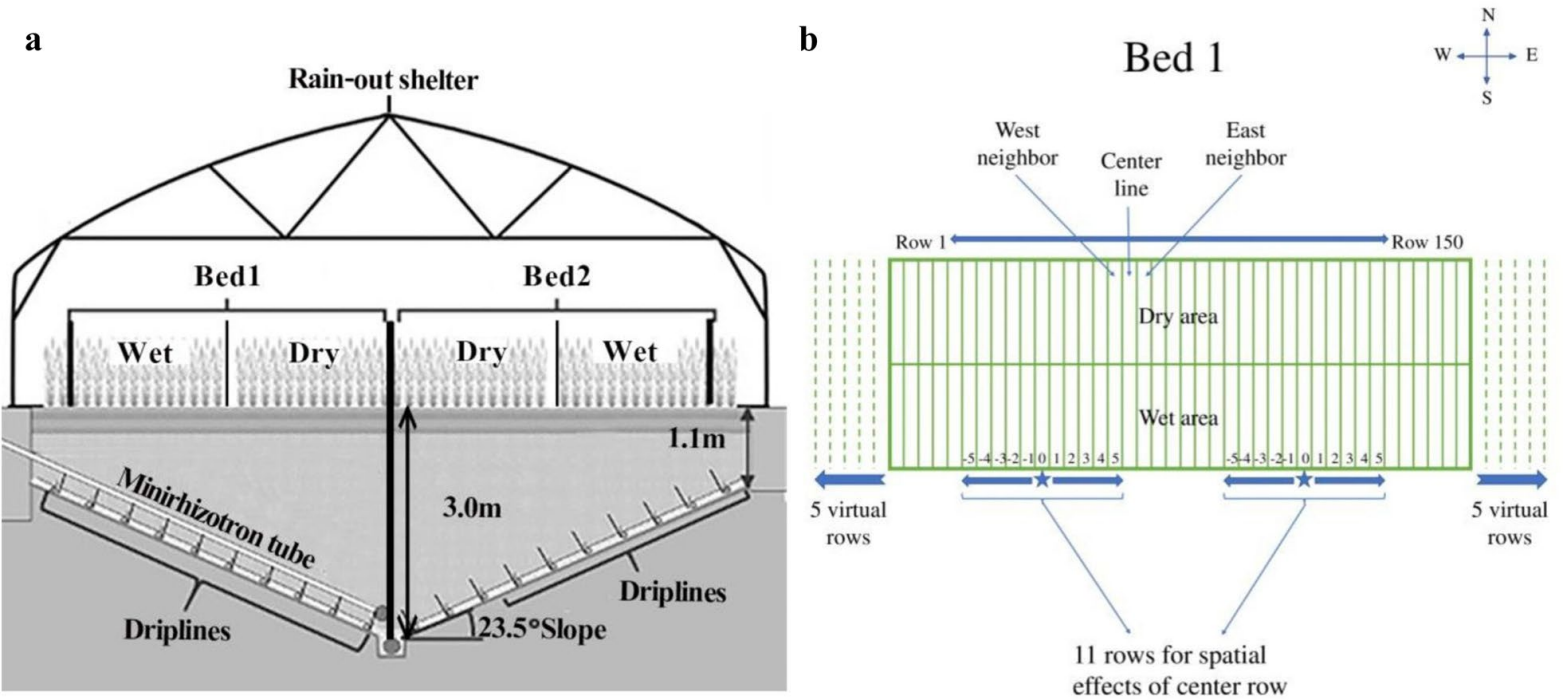
Cross-section of two beds in semi-field facility and vertical view of one bed [6, 31]. **a** is cross-section of two beds; **b** is vertical view of one bed

Wheat, one of the most important food crops, is widely grown across different regions in the world [1]. To meet the demand of a growing human population, an intensification of agriculture, which allows greater yield to be obtained from existing farmland with sustainable inputs of water and fertilizer, is needed [2, 3]. Many of the constraints on yield in agricultural systems have been found in the root system of crops [4]. However, the investigation of the root system is difficult because of the complexity of soil environments compared with laboratory conditions [5]. To investigate the deep root system in a non-laboratory condition as well as to obtain direct and stable measurements, a semi-field root phenotyping facility has been constructed recently [6]. A semi-field system is defined as a closed environment presenting all features necessary for crop life cycle completion, within which the natural ecosystem is under control while still exposed to ambient environmental conditions [7]. Figure 1a shows one part of the semi-field root phenotyping facility including two experimental beds, among which one was equipped with minirhizotrons. This facility has a capacity of 150 rows (Fig. 1b) in each of eight experimental beds so that relatively large plant populations could be sown and studied. For practical purposes, every two independent experimental beds were built next to each other. The facility is designed for both direct phenotyping of root traits through minirhizotrons and allow testing different of genotypes based along a water stress gradient.

Plant breeding has been successful in increasing the yield of most major crops [8]. This has contributed to a steady increase in cereal production without a large increase in acreage devoted to the production [8]. This significant progress has been driven by the extensive research in developing and establishing new breeding technologies and rapid uptake of such new technologies by breeding programs [9]. A strategy suggested to improve breeding has been the utilization of molecular markers in breeding programs through marker-assisted selection (MAS) [10]. The use of genome-wide association studies (GWAS) combined with subsequent MAS has been shown as an effective tool in breeding program for qualitative traits under simple Mendelian genetic control [11]. For complex polygenic quantitative traits, e.g. grain yield, the application of MAS did not provide considerable genetic improvement. Instead, the implementation of genomic selection (GS) has been studied and shown as a promising method to be applied in the breeding program for polygenic traits [12].

In GS, a large set of markers are simultaneously incorporated into a model in order to obtain genomic prediction (GP) of genomic expected breeding values (GEBVs) [12]. The efficiency of a breeding program can be improved and the cost of resources can be reduced by applying GP to predict GEBVs of individuals for selection as early as possible [13]. The accuracy of GP in wheat has been investigated in several recent studies, in which different population sizes and various models/methods were compared [9, 14–17]. Many factors can affect the prediction accuracy in GP, for example, size of training data, relatedness between training and test individuals, cross-validation strategies, marker density, as well as the model/method used for prediction of GEBVs [18, 19].

One of the most popular statistical models used for prediction of GEBVs is genomic best linear unbiased prediction (GBLUP), which integrates the genomic relationship matrix (**G**) constructed from all available markers [20]. Based on the GBLUP model, various model extensions have been proposed and investigated. For example, in addition to additive genetic effects, non-additive genetic effects such as dominance and epistatic effects can also be included in the model [21]. Another example is modeling of genotype-by-environment interactions [22].

Modeling of interaction between individuals is yet another kind of model extension. The interaction between individuals was demonstrated in animals in respect to social interactions. With animals, these interactions can be social interaction in a group, while with plants they can be physical due to competition for limited recourses. A previous study showed that interaction among individuals could create substantial heritable variation [23]. The methods presented in this previous study [23] were applied to a population of layer chickens with high mortality due to pecking behavior and estimates of genetic parameters for survival days was obtained [24]. The results showed that two-thirds of the total heritable variation was hidden to classical analysis due to social interactions [24]. This interaction among individuals also been known as indirect genetic effects (IGEs), which arise whenever genetic effects expressed in one individual influence the phenotype of a social partner [25], have been shown as important to trait variation. For example, it has been shown that IGEs might lead to phenotypic and genotypic evolution moving in different directions [26].

In practice, micro-environments differ across a field, and neighboring individuals can have similarity in their phenotypic performance because of the sharing of micro-environment [27]. Therefore, some studies investigated the spatial effects in linear mixed model by fitting the spatial effects using different strategies [27–29]. Another issue which should be considered when plants are grown in an individual row field trial is the influence from the neighboring individuals. This can be through competition for resources, such as water and nutrients from the soil, and light capture above ground [30]. The IGEs from neighbors can be substantial for plant growth. It was shown that the inclusion of such effects from neighbors could decrease error in estimation of genetic effects [30]. However, to our knowledge, the study of neighbor effects using genomic information is very rare.

The objectives of this study were to (1) investigate genetic variation in grain-related and deep root traits in winter wheat; (2) study genomic by water availability interaction; (3) quantify neighbor effects when lines were grown in adjacent rows; and (4) analyze the possibilities of predicting breeding values of new wheat lines based on genomic prediction from the semi-field root phenotyping facility.

## Results

In this study, four grain-related traits: grain yield (GY), thousand-kernel weight (TKW), protein concentration (PC), and total nitrogen content (NC), and two root traits were total root length between 1.2 and 2 m depth (TRL) and root length in four intervals on each tube (IRL). Based on the grain-related and root data collected from a semi-field phenotyping system, variance components (VCs) for each trait were estimated and the accuracy in genomic prediction of breeding values (ACC) were assessed by cross-validation. Two sets of models, with or without the effects of neighbors on both sides of each row were applied. The first model (GM1) allocated the phenotypic variation into direct genetic and environmental components, while the second model (GM2) allocated the phenotypic variation into an indirect genetic component from the neighbors on both sides in addition to the effects included in the first model.

### Descriptive statistics

Table 1 gives the descriptive statistics of all the traits analyzed in this study. As shown in Table 1, the number of observations for grain-related traits were from 1043 to 1045. Some records were missing compared with the potential of 1200 (four experimental beds, two harvesting areas/bed and 150 rows/bed) records, due to failure in growth or seeding errors in these rows. The average of GY, TKW, PC and NC, respectively, were 7.44 t ha^−1^, 39.99 g, 11.49% and 135.82 kg N ha^−1^. For root traits, there were 255 records for TRL and 986 records for IRL. The average of TRL was 99.79 cm, and the average for IRL was 25.81 cm.

**Table 1:** Descriptive statistics for all the traits

Type	Trait	Unit	No. of records	Average	S.D.	Min	Max
Grain-related	GY	t ha^−1^	1043	7.44	1.60	1.52	12.71
	TKW	g	1044	39.99	3.82	25.47	50.35
	PC	%	1045	11.49	0.93	9.41	15.50
	NC	kg N ha^−1^	1043	135.82	26.72	28.70	218.07
Root	TRL	cm	255	99.79	37.13	16.21	198.59
	IRL	cm	986	25.81	20.01	0.01	80.46

GY is grain yield, TKW is thousand-kernel weight, PC is protein concentration, NC is total nitrogen content, TRL is total root length between 1.2 and 2 m depth, IRL is root length in four intervals on each tube

### Estimation of variance components

**Fig. 2:**
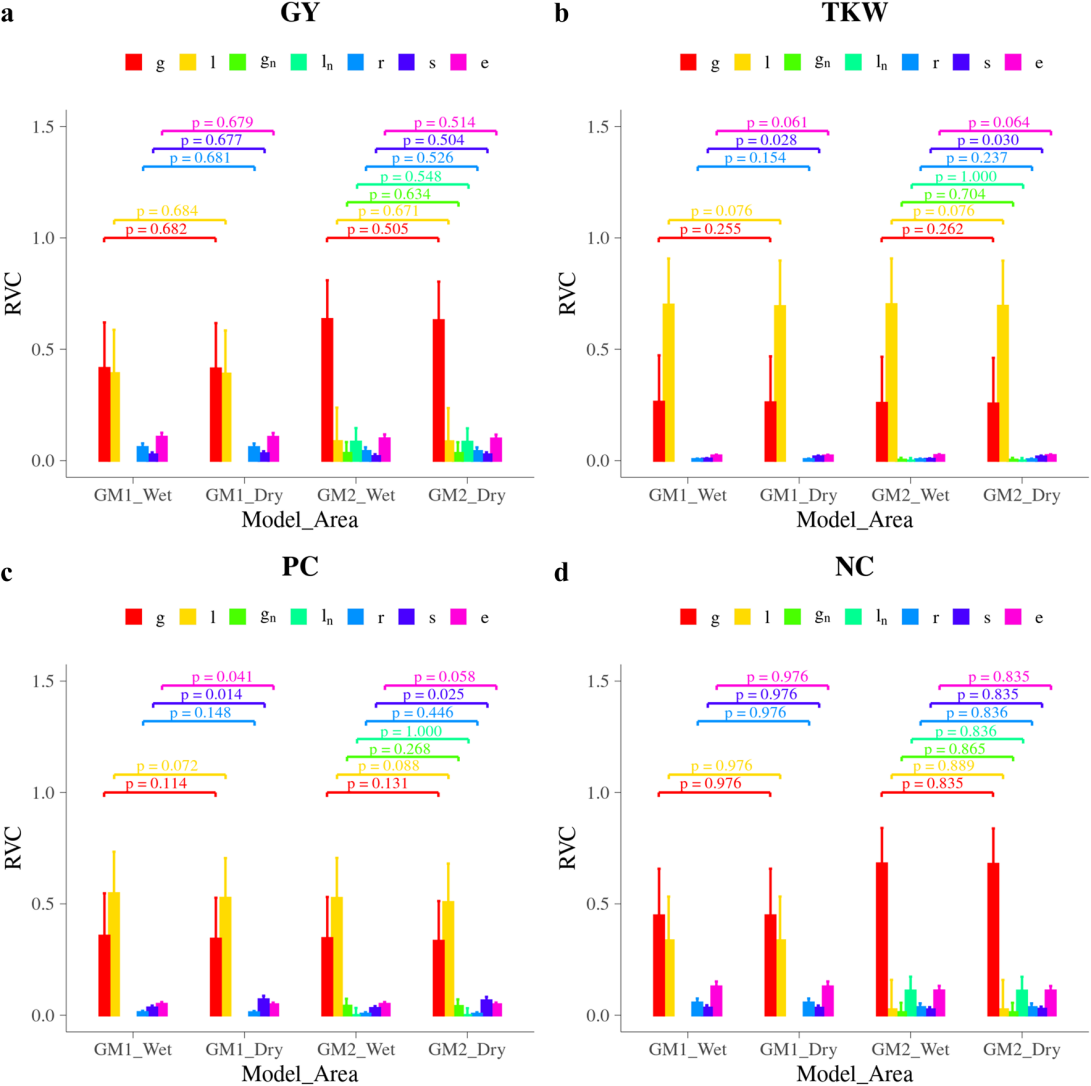
Estimated relative variance components for grain-related traits in different models and wet or dry parts. **a** is grain yield (GY); **b** is thousand-kernel weight (TKW); c is protein concentration (PC); and d is total nitrogen content (NC). *g* is additive genomic effects; *l* is line effects; *g*_*n*_ is additive genomic effects for neighbors; *l*_*n*_ is line effects for neighbors; *r* is row effects; *s* is spatial effects; *e* is residual effects. y-axis is relative variance components (RVC); x-axis is the model used and the area records obtained from; p values are for comparisons between wet and dry areas for each effect

The models used separated total phenotypic variance into variance components (VCs) due to additive genetic effects, non-additive genetic effects, additive genetic effects of neighbors, non-additive genetic effects of neighbors, row effects, spatial effects over the facility separately for each wet and dry harvesting area, and finally error variance. The estimated VCs were expressed as relative variance components (RVCs), which were the proportion of each VC that contributed to the total phenotypic variance among lines. The RVCs for grain-related traits are shown in Fig. 2. For each grain-related trait, two different models with neighbor effects (GM2) or without neighbor effects (GM1) were applied. The proportion of each VC was calculated for the wet and dry areas separately due to the heterogeneous spatial variance in wet/dry recording areas.

Averaged over wet and dry areas, the RVC of genomic effects (***g***) when using GM1 which is equivalent to estimated narrow sense heritability ($\widehat{{h^{2} }}$), were 0.414 for GY, 0.263 for TKW, 0.350 for PC and 0.447 for NC. For PC, $\widehat{{h^{2} }}$ in the wet area was around 3.8% larger than in the dry area (p = 0.114). For other grain-related traits, $\widehat{{h^{2} }}$ were more similar between wet and dry areas. In GM2, inclusion of neighbor effects increased the $\widehat{{h^{2} }}$ for GY and NC. Averaged across two areas, the $\widehat{{h^{2} }}$ increased 0.219 for GY and 0.233 for NC.

The RVC for line effects (***l***) were 0.391 for GY, 0.697 for TKW, 0.537 for PC, and 0.335 for NC, averaged across wet and dry areas by using GM1. For PC, the RVC for ***l*** in the wet area was 4.00% larger than in the dry area (p = 0.072). The RVC for ***l*** were similar between two areas in other grain-related traits. Significant change when using GM2 was also observed in the RVC of ***l*** for GY and NC. When including neighbor effects in the model, RVC for ***l*** decreased to 0.086 for GY and to 0.026 for NC.

The sum of RVCs for genomic effects (***g***) and line effects (***l***) is equivalent to estimated broad sense heritability ($\widehat{{H^{2} }}$), were 0.805 for GY, 0.958 for TKW, 0.886 for PC and 0.783 for NC. Similar to $\widehat{{h^{2} }}$, $\widehat{{H^{2} }}$ in the wet area was around 3.9% larger than in the dry area for PC (p = 0.027). For other grain-related traits, $\widehat{{H^{2} }}$ were more similar between wet and dry areas. Whereas in GM2, including of neighbor effects did not increase the $\widehat{{H^{2} }}$ for all grain-related traits, which were 0.718 for GY, 0.955 for TKW, 0.855 for PC, and 0.705 for NC averaged across two areas.

The RVC for row effects (***r***) were relatively small compared with genomic effects (***g***) and line effects (***l***), the average of two areas using GM1 were 0.060 for GY, 0.006 for TKW, 0.013 for PC, and 0.056 for NC. There was also a trend of decreasing the RVC of ***r*** in GY and NC when including neighbor effects in the model (GM2).

When using GM1, the RVCs due to spatial effects (***s***) were 0.027 for GY, 0.008 for TKW and 0.034 for PC in the wet area, compared with wet area, they were 0.032 (GY, p = 0.677), 0.018 (TKW, p = 0.028) and 0.071 (PC, p = 0.014) in the dry area, i.e. the spatial variances were different in the wet and dry harvesting areas for TKW and PC. The RVC due to ***s*** for NC was same for both wet and dry areas. A tendency of a decrease in RVC of ***s*** was also observed in GY and NC when including neighbor effects in the model (GM2).

The RVC due to residuals (***e***) were quite similar and stable, compared with other effects, between wet and dry areas as well as different models. When using GM1, the RVCs for ***e*** were 0.106 for GY, 0.023 for TKW, 0.050 for PC and 0.128 for NC. The inclusion of neighbor effects did not change the RVC for ***e*** for TKW and PC, while for GY and NC the RVC of ***e*** decreased.

Consistent with the significant change of RVCs in GY and NC when using GM2, a higher proportion of genomic effects for neighbor (***g***_***n***_) and genomic effects for line (***l***_***n***_) was observed in these two traits compared with TKW and PC. The RVC of ***g***_***n***_ were 0.034 for GY and 0.013 for NC, and the RVC of ***l***_***n***_ were 0.084 for GY and 0.110 for NC. The neighbor effects were small in TKW and PC. The differences in RVCs of all the common effects (***g***, ***l***, ***r***, ***s***, ***e***) between wet and dry areas were similar between GM1 and GM2.

**Fig. 3:**
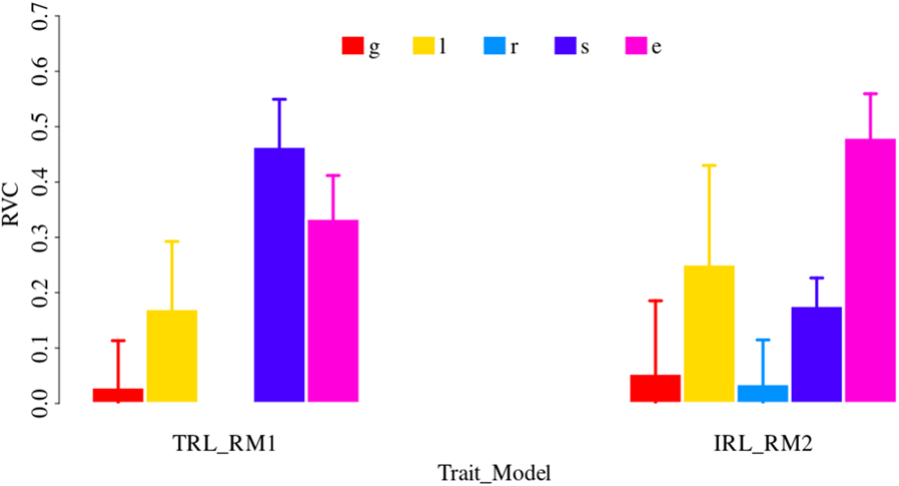
Estimated relative variance components for root traits. *g* is additive genomic effects; *l* is line effects; *r* is row effects; *s* is spatial effects; *e* is residual effects. y-axis is relative variance components (RVC); x-axis is trait; TRL is total root length between 1.2 and 2 m depth, IRL is root length in four intervals on each tube

Estimated RVCs for each line in root traits are shown in Fig. 3. Due to model complexity, only the models without neighbor effects (RM1 and RM2) were applied for root traits. For each minirhizotron tube, there was one TRL record while there were four repeated IRL records. In addition to the effects accounted in RM1 for TRL, RM2 was applied to IRL to account for the fixed depth interval effects and random row effects. Compared with grain-related traits, the $\widehat{{h^{2} }}$ were small in root traits. The $\widehat{{h^{2} }}$ were 0.030 for TRL and 0.055 for IRL, the RVCs of line effects (***l***) were 0.171 for TRL and 0.252 for IRL. To sum up RVCs for genomic effects (***g***) and line effects (***l***), the $\widehat{H}^{2}$ were 0.201 for TRL and 0.306 for IRL. However, the RVCs for spatial effects (***s***) and environmental effects (***e***) were large in both root traits. The RVCs for ***s*** were 0.464 for TRL and 0.177 for IRL, and the RVCs for ***e*** were 0.334 for TLR and 0.481 for IRL. For IRL, the effects of row (***r***) accounted for 0.036 of the total phenotypic variances.

**Fig. 4:**
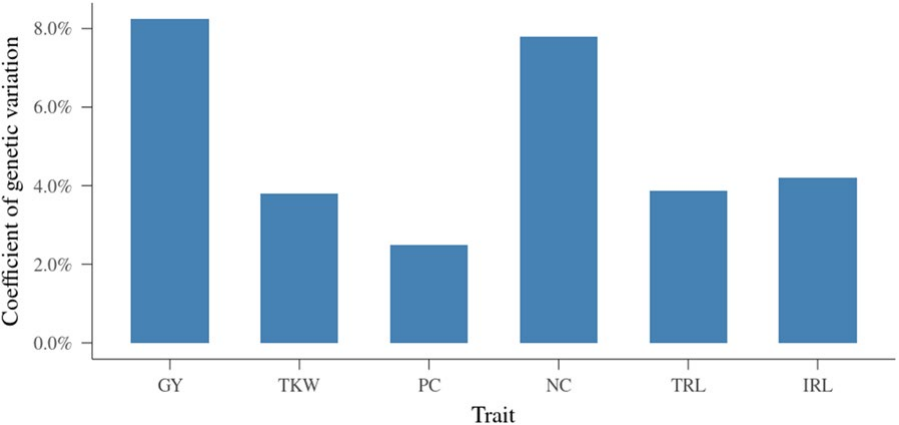
Coefficient of genetic variation for grain-related and root traits. y-axis is coefficient of genetic variation; x-axis is trait; GY is grain yield, TKW is thousand-kernel weight, PC is protein concentration, NC is total nitrogen content, TRL is total root length between 1.2 and 2 m depth, IRL is root length in four intervals on each tube

In addition to the estimation of VCs, coefficients of genetic variation (***c***_***v***_), calculated as the ratio between square root of variance explained by additive genomic effects (***g***) and the mean value of observations, were also computed and the results are presented in Fig. 4. In order to compare grain-related and root traits, only the results from models without neighbor effects are reported here. For grain-related traits, the ***c***_***v***_ were 8.24% for GY, 3.81% for TKW, 2.49% for PC and 7.80% for NC. The ***c***_***v***_ for root traits were 3.88% for TRL and 4.20% for IRL. Even though the $\widehat{{h^{2} }}$ of root traits were low compared with grain-related traits, the ***c***_***v***_ of root traits were higher than for TKW and PC.

### Genomic prediction and cross-validation

**Fig. 5:**
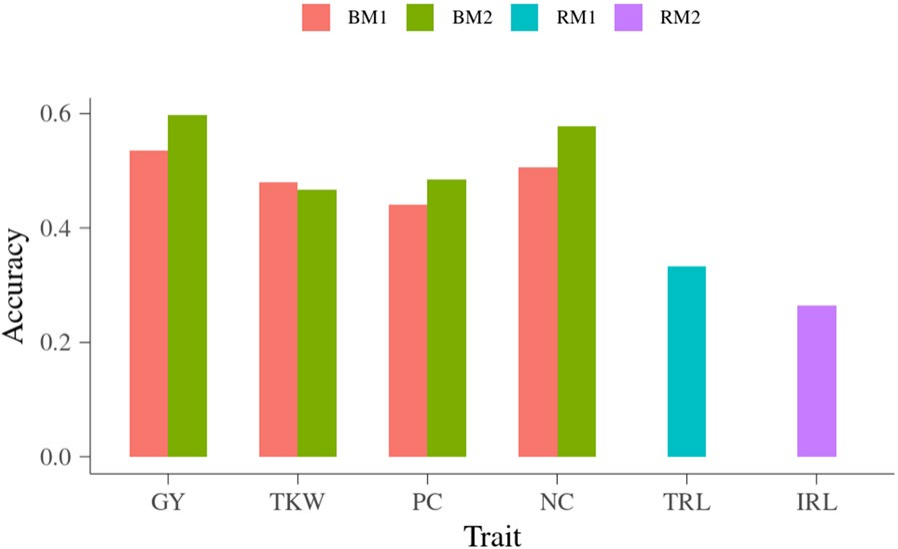
Accuracy of prediction for each line in grain-related and root traits. y-axis is accuracy of prediction; x-axis is trait; GY is grain yield, TKW is thousand-kernel weight, PC is protein concentration, NC is total nitrogen content, TRL is total root length between 1.2 and 2 m depth, IRL is root length in four intervals on each tube

Leave-one-line-out cross-validation (LOO-CV) was conducted for models with or without neighbor effects. In each round of the cross-validation, the phenotypic records of one line were left out as validation dataset while the rest were used as training dataset to estimate model parameter and to obtain the predictions of genomic expected breeding values (GEBVs) for the left-out line. After all the validation rounds, the predictions of GEBVs for all lines were obtained. The accuracy of prediction of GEBVs (ACC) for each line in grain-related and root traits are presented in Fig. 5. This is the accuracy of predicting a genotyped line that does not have own phenotypic records but only genomic information. When using GM1, the ACC was 0.535 for GY, 0.480 for TKW, 0.440 for PC, and 0.505 for NC. Considering neighbor effects in the model significantly increased the ACC of direct breeding values for GY, PC and NC, which were 0.598 (GY), 0.485 (PC) and 0.578 (NC) when using GM2. Compared with GY, PC and NC, the ACC of TKW, which was 0.467, did not increase when using GM2. When using models without neighbor effects, the ACC for TRL was 0.334, and for IRL ACC was 0.264.

**Fig. 6:**
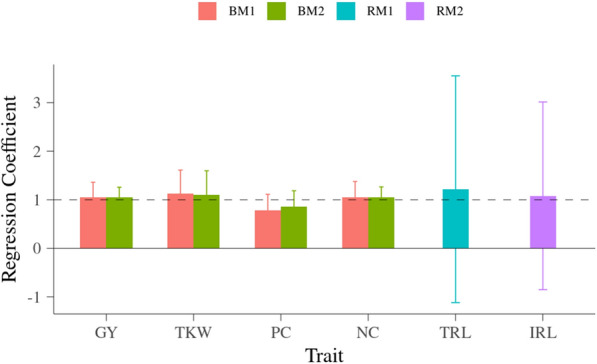
Inflation of prediction for each line in grain-related and root traits. y-axis is regression coefficient of $\overline{y}_{c}$ on $\widehat{g}$; x-axis is trait; GY is grain yield, TKW is thousand-kernel weight, PC is protein concentration, NC is total nitrogen content, TRL is total root length between 1.2 and 2 m depth, IRL is root length in four intervals on each tube; horizontal dashed line is regression coefficient of 1, which is the situation of no inflation, horizontal solid line is regression coefficient of 0

The potential inflation of predictions for each line in grain-related and root traits are presented in Fig. 6. When using GM1, the regression coefficients were 1.045 for GY, 1.122 for TKW, 0.788 for PC and 1.054 for NC. When using GM2 by considering neighbor effects, the regression coefficients were 1.072 for GY, 1.094 for TKW, 0.855 for PC and 1.078 for NC. The inflation of prediction from GM1 and GM2 were similar. For root traits, the regression coefficients were 1.218 for TRL and 1.082 for IRL. The regression coefficients for all the grain-related and root traits were not significantly different from unity, i.e. there were no significant inflation in the predicted genomic breeding values.

## Discussion

In this study, traits related to grain yield and root length of winter wheat measured in a semi-field root phenotyping facility [6] were analyzed. Variance components were estimated and genomic predictions were explored by using models with or without effects of neighboring genotypes.

The models used in the current study did not include genomic by water availability area (wet and dry) interaction effects since preliminary investigation showed that the proportion of variance due to genomic by water availability were close to zero. Some previous studies also showed that there was no line by water availability area interaction [31, 32]. One of the reasons could be the design of the water availability gradient. The observations of grain-related traits were obtained for wet and dry areas by dividing the row into two equal sized areas that were harvested separately. The division of the water availability gradient created two areas including a wet area with a short distance to water and a dry area with long distance to the sub-surface watering system. This gradient of water availability could reduce the variation and cause difficulty in detecting genomic by water availability since wet area had much less soil mass and thus less access to nutrients than the dry area. Other factors might influence the lack of subsurface irrigation response, and it has been speculated that a lack of nutrients will lower growth potential when water is supplied to a nutrient poor subsoil, and do not provide water to the nutrient rich topsoil being dry at all locations across the water availability gradient [31]. Therefore, for the current study, genomic by water availability effects were excluded in the models.

### Estimates of variance components and heritabilities

There were four grain-related and two root traits analyzed in this study. The proportion of total phenotypic variance accounted for by genomic effects (***g***), corresponding to narrow sense heritability (*h*^*2*^), was estimated. Using different models, the estimates of *h*^*2*^ of GY ranged from 0.41 to 0.67. The estimates were higher than the *h*^*2*^ reported from some previous studies using phenotypic records only [33, 34]. One of the studies on winter wheat GY reported *h*^*2*^ estimates, which ranged from 0.26 to 0.75, for 12 F_3_ wheat populations [35]. The *h*^*2*^ estimate of TKW was estimated as 0.26 from both models. The *h*^*2*^ of TKW reported from previous studies were similar or higher than in the current study, e.g. 0.32 [36] and 0.57 [37]. One study using genomic data obtained *h*^*2*^ estimated as 0.52 [38], which is also higher than in the current study. The reason could be the inclusion of line effects (***l***) in the current models while in the previous study [38] ***l*** were not included due to lack of replication. Such models may lead to biased estimates of additive genetic variance due to non-additive genetic effects. The estimates of *h*^*2*^ were around 0.35 for PC using both models. Compared with 0.51 reported in a previous study [38], the lower estimates in the current study can also be caused by fitting of ***l*** as discussed earlier. Estimated *h*^*2*^ for NC range from 0.45 to 0.72 using different models in the current study. One previous study showed that the broad sense heritability (*H*^*2*^) of NC as 0.31 in which 31% of the genetic variance was contributed by genotype and 66% was contributed by genotype by year interaction effects [39].

The estimates of relative variance components (RVCs) for line effects (***l***) varied from 0.34 to 0.70 for grain-related traits when using GM1, and from 0.03 to 0.70 when using GM2. The variances of ***l*** include the part of the genetic variance, which cannot be explained by genomic markers as well as non-additive genetic effects. The effects due to common area and origin of the seeds used when establishing the experiment could also be included into the variances of ***l***. Decreasing RVCs for ***l*** when including neighbor effects in the model, for GY and NC, also suggested that the variances of ***l*** were affected by variances from other sources e.g. neighbors. In the ideal randomization of the experiment, each line will not have the same neighbor twice, to prevent the variance of ***l*** including variances from neighbors, but in the current study, the randomization could be one of the reasons for this issue. Some preliminary studies were done by comparing models including ***l*** or not, from which the results suggested that the exclusion of ***l*** can create more inflation in genomic predictions (results not shown) indicating poorer fit for such a model. Therefore, the models reported in the current study always considered the effects of ***l***. The sum of RVCs for genomic effects (***g***) and line effects (***l***), the estimates of broad sense heritabilities (*H*^*2*^) were obtained, which varied from 0.78 to 0.96 for grain-related traits when using GM1, and from 0.71 to 0.95 when using GM2. The estimate of *H*^*2*^ represents the total genetic variation in a trait, except for the additive genetic variances explained by genomic information, it can also include the additive genetic variances not explained by markers and the non-additive genetic variances. When using GM1, the estimates of *H*^*2*^ were much larger than *h*^*2*^ and the differences ranged from 1.7 to 3.7 times but when using GM2, the ratio between *H*^*2*^ and *h*^*2*^ decreased to a range of 1.0 to 3.7. The high level of *H*^*2*^ suggested that the grain-related traits were under a high degree of genetic determination.

Spatial effects (***s***) were modeled in this study and the estimates of RVCs for spatial effects ranged from 0.01 to 0.07 for grain-related traits with both wet and dry areas and in both GM1 and GM2. Modeling neighbor effects did not affect the proportion of variances caused by ***s***. The modeling of ***s*** assumed a heterogeneous variance for the two water availability areas separately, since preliminary results showed that the pattern and the degree of ***s*** were different when analyzing the data within each water availability area. Spatial effects were investigated by other studies in different ways. For example, considerable spatial variation was found in wheat data [28]. In their study, spatial variation was corrected by introduce of a covariable, which was calculated as the value of phenotypic in each plot minus mean phenotypic value of neighboring plots. This introduces a regression of a function of the data that might lead to underestimation of total phenotypic variances. In the current study, this was avoided by modeling the spatial effects as a running sum of random effects due to rows surrounding each plot. Another discovery from the current study was that a more pronounced spatial variation was observed under dry areas, and led to a lower *h*^*2*^ than the wet areas.

Neighbor effects were considered by applying GM2 and both genomic effects for neighbors (***g***_***n***_) and line effects for neighbors (***l***_***n***_) were modeled. The estimates of RVCs for ***g***_***n***_ ranged from 0.003 to 0.042, and ranged from 0 to 0.110 for ***l***_***n***_. Modeling of neighbor effects led to significant change of variance structures for both GY and NC, whereas the changes in TKW and PC were small. For both GY and NC, the RVCs accounted by genomic effects (***g***) increased considerably but the line effects (***l***) decreased in the same amount. This indicates that if neighbor effects are excluded from the model a large part of these effects are picked up by the effects of ***l***. In a study for cassava data [40], neighbor effects were investigated as competitive ability. From their simulation study, accuracy in estimating genotypic effects increased when including competitive effects in the model. Through their analysis for nine trials and four traits, significant competition effects were observed for 12 of the 36 combinations [40]. In plants, genotype response to a studied specific stress can be complicated by competition effects from neighbors [30]. The neighbor effects reported in the current study were a type of indirect genetic effects, which are mostly known as social genetic effects in animal breeding. The social genetic effects can be positive, for which mothering behavior is one example, or negative such as competition and aggression. A previous study reviewed the social genetic effects in animal breeding, and it showed that in most of the animal populations, there are substantial social (indirect) genetic effects [41]. In animals, the social genetic effects come from the *n*-*1* other individuals in a group of *n* individuals, and the magnitude of social effects may depend on group size [41]. In the current study, since the lines were planted in rows, the indirect genetic effects were modeled as the neighbor effects from both sides of the row studied.

For the two root traits analyzed in this study, the estimates of *h*^*2*^ ranged from 0.03 to 0.05, whereas the proportion of other effects were generally larger than *h*^*2*^, e.g. the effects of line accounted for 0.17 to 0.25 of total phenotypic variances. Though *h*^*2*^ estimates for root traits were low compared with grain-related traits analyzed in this study, relatively, there were the same amount of genetic variation in the root traits. The coefficients of genetic variation (***c***_***v***_) of root traits were even higher than TKW and PC, which suggested that the amounts of genetic variation for root traits were comparable with genetic variation for the yield related traits. However, the root measurements were dominated by other effects e.g. spatial effects and measurement errors, which caused the limited *h*^*2*^ obtained for root traits using the current phenotyping methods for root length. The sum of RVCs for additive genetic effects (***g***) and line effects (***l***) shows the broad sense heritabilities (*H*^*2*^), which varied from 0.20 to 0.31 for root traits. The estimates of *H*^*2*^ were 5.6 to 6.8 times larger than *h*^*2*^ and the differences between *H*^*2*^ and *h*^*2*^ in root traits were even larger than in grain-related traits. The degree of *H*^*2*^ suggest that the root traits are under a considerable degree of genetic determination even though only a small proportion can be explained by genomic information. In addition, the current number of records also limited model complexity for these two traits. To reduce environmental noise in future experiments the number of root imaging campaigns should be increased and practices to reduce spatial effects within the facility should be implemented.

The genotype by environment interaction caused by water availability was investigated in the current study, but no interaction was found. This interaction might be better investigated by changing the strategy of harvesting, for example harvest the plants in smaller unit instead of half row each time, which allow for more observations to be obtained along with the water gradient. Models that better fit the observations could be expected when the plants been harvested in smaller blocks. Large environmental variances were observed in root traits and led to the relatively small genetic RVC. To solve this problem and reduce the influence from non-genetic effects, the precision of the root imaging should be increased.

### Genomic prediction and validation

Both accuracy for prediction of GEBVs (ACC) and inflation of predictions were investigated in this study for all grain-related and root traits, and the results from models with or without neighbor effects were compared.

The ACC were measured as the correlation between corrected phenotypic values (***y***_***c***_) and GEBVs and then scaled by square root of heritability. This corresponds to the correlation between predicted and true breeding values [42]. The ACC ranged from 0.440 to 0.535 for the four grain-related traits when using GM1, and from 0.467 to 0.598 when using GM2 including neighbor effects. The ACC showing the accuracy of predicting the underlying true additive genetic value, clearly shows the power of GP utilizing information from related individuals to predict the additive genetic value of specific lines. Inclusion of neighbor effects significantly improved the ACC for GY and NC. The increase in ACC for GY and NC were consistent with the marked change in the VCs pattern when including neighbor effects. Improvement of ACC when modeling neighbor effects were also found in the previously mentioned study for cassava where up to 25% increase in accuracy was observed [40]. The ACC obtained in this experiment was expected to be relatively low due to the small size of the population used to train the models.

In a previous study, it was reported that predictive ability (PA), which was the correlation between corrected phenotypic values (***y***_***c***_) and GEBVs, for TKW ranged from around 0.26 to 0.50 (corresponding as 0.36 to 0.69 in ACC) by using two different validation schemes [38]. In this previous study, tenfold cross-validation, where the whole population was randomly divided into 10 random groups, gave higher PA than the leave-set-out cross-validation (LSO-CV), where each set was defined as one of the breeding cycles included in the study. Relatively larger PA compared with the current study could be explained by the materials used for the previous study was 1152 F6 winter wheat lines from four different breeding cycles, which was much larger than in the current study, i.e. the increased accuracy can be explained by the larger training population. Effect from size of training dataset on the genomic prediction accuracy was investigated in the previous studies [16, 17]. For example, in the study for 309 spring barley lines, it was concluded that using less than 200 lines in the training set could result in low PA [16]. Another study explored a total of 988 advanced wheat breeding lines for yield, lodging, and starch content, and the results showed that training population of around 700 lines were enough to yield the highest observed predictive abilities [17]. In the current study, only 84 lines were involved in the cross-validation procedure. Considering this, the degree of PA was reasonable, but higher PA could be expected by increasing the number of lines included in the experiment.

Compared with TKW, the ACC obtained for PC were a bit higher. The PA for PC was also investigated in previous study [38]. The authors reported that in the tenfold cross-validation scheme, the PA for PC were also bit higher than for TKW, however, the PA for PC obtained from the LSO-CV scheme were lower than TKW. The PA of PC reported from another two studies were above 0.6 by using models integrating genomic information, which merged the merits of genomic selection with phenotypic selection in preliminary GY trials [43, 44].

The ACC of NC were very close to the results for GY, and were 0.505 using GM1 and 0.578 using GM2. One of the reasons could be that the NC was computed from grain GY and grain PC concentration. NC is required to maintain active photosynthesis in the canopy to support GY and PC [45]. Grain NC is a reflection of nitrogen use efficiency, which could increase GY at a given level of nitrogen fertilizer [39]. The moderate ACC obtained for NC can ensure a reasonable genetic progress when breeding for grain NC and facilitate the breeding for nitrogen use efficiency.

For root traits in the present study, the ACC were 0.264 to 0.334, which was consistent with the lower estimates of VC due to additive genetic effects. Though the ACC were lower than for grain-related traits, it was clearly shown that reasonable accuracies could be obtained for the studied root traits in the current phenotyping facility.

The accuracy obtained for genomic predictions of additive genetic effects were as expected for both grain-related and root traits since a relative limited training population were used in this study. Previous studies clearly showed that increasing the training population significantly improve the prediction accuracy [16, 17].

### Perspectives in practical plant breeding

Crops with deep roots have the ability to access more water and nutrients in the soil. Continuing climate change is expected to increase the frequency of periods of limited water supply, so that crops often would experience drought during growth [46]. With deeper roots, plants can uptake potential resources in deeper soil to ensure the plants having better growth and higher yields. Therefore, breeding of plants with deeper roots has the potential to ensure greater resilience towards drought in future crops. The semi-field facility combined with genomic prediction of deep root traits presented in the current study provided the opportunity to investigate the possibility of breeding for deeper root and better production. This facility can overcome problems caused by the difference between field conditions and laboratory conditions, and root growth can be observed while the plant growing in the soil. The results from the current study clearly show the possibility of genomic prediction in grain-related and deep root traits. Selection of lines based on genomic prediction can shorten the breeding cycle and decrease the cost in breeding programs. The models applied in the current study provide the possibility to investigate the indirect genetic effects through accounting for the effects from neighbor lines. This is important for the strategy where crops been planted in rows. The genetic correlation between direct genetic effects and indirect genetic effects could be investigated in a larger experimental design in the future. In the current study, we assumed that direct and indirect genetic effect were independent due to the limited amount of lines included in the experiment. With genomic information and appropriate methods/models, it is possible to select lines with better yield and drought resistance before being planted in the field.

## Conclusions

In this study, genomic prediction was carried out for grain-related and root traits of winter wheat measured in a semi-field root phenotyping facility. Phenotypic and genotypic data were analyzed for 84 winter wheat lines. Two sets of models were used in order to study consequences of including neighbor effects in the model. We report estimated variance components and performance of genomic prediction using a leave-one-line-out cross validation strategy.

Results showed that the grain-related traits, GY, TKW, PC, and NC had high to moderate narrow sense heritabilities. For root traits, heritabilities were low. However, the relative amount of additive genetic variation for deep root traits were of similar magnitude as for the grain-related traits but the low heritabilities were caused by very large environmental variance due to spatial effects and measurement errors.

In no case did we find any genotype by water availability interaction, which could be due to the water availability design in this study, which was a gradient and this might weaken the power to detect the genotype by water availability interaction.

For the above ground grain-related traits, we found effects of neighbors. The effects were generally small, but including neighbor effects increased the estimated additive genetic variance of the traits and increased accuracy of predicting breeding values for lines without own phenotypic records.

For both grain-related and root traits it was possible to obtain genomic predictions of additive genetic effects with an accuracy as expected based on the limited training population available.

Especially the root traits had large environmental variance due to large spatial effects and measurement error. More accuracy in the methods for phenotyping deep root length are needed to obtain accurate genomic predictions.

## Methods

### Plant material

For this study, 84 winter wheat (*Triticum aestivum L.*) lines with 15 K SNP genotypic data (Additional file 1: Table S1) were selected. The lines included high yielding northern, and central European varieties and advanced breeding lines from Sejet Plant Breeding and Nordic Seed. These winter wheat lines were sown in a semi-field root phenotyping facility [6] having a capacity of 150 rows per experimental bed. The novel phenotyping facility was named “RadiMax” and developed in order to study the root growth and soil resource acquisition under semi-field conditions. In total, the facility covers 1600 m^2^ for all the beds constructed. The facility allows roots to be observed in the 0.7–2.8 m soil depth interval through the minirhizotrons using a multispectral imaging system. A total of four experimental beds were used for the study of winter wheat. The beds were constructed in pairs with that were operated independently with 150 rows in the north side and 150 rows in the south side of the construction. Along each row, a water stress gradient was created by a multi-depth sub-irrigation system and movable rainout shelters. Figure 1a shows a part of the semi-field root phenotyping facility including two experimental beds, of which one was equipped with minirhizotrons. The bottom of facility is sloped allowing for the sub-irrigation system to be applied and this induced the water gradient. Among the four experimental beds for winter wheat, two were equipped with minirhizotron along the sloped bottom. In each of the four beds, there were an equal number of rows (150, Fig. 1b). The experiment had four replicates with one replicate in each experimental bed, so there were 600 experimental rows in total. For practical reasons harvesting were carried out in two areas of each row due to limitations in the harvesting tools. This harvest strategy split each experimental bed into two areas from the middle. A wet area with short distance to the water supply and a dry area with a longer distance to the water supply as shown in Fig. 1. Therefore, for the grain-related traits, potentially, there could be 1200 records (four beds, two harvesting area for each bed, 150 rows for each bed) available for analysis.

Four grain-related traits (Additional file 1: Table S2) were analyzed in this study: grain yield (GY), thousand-kernel weight (TKW), protein concentration (PC) and total nitrogen content (NC). For these traits, each row was split into wet and dry areas and harvested separately. Therefore, potentially, 1200 observations were available, with four beds, 150 rows within each bed, and within each row, a wet and a dry area. GY was calculated based on the respective area of each sample and the dry grain weight and expressed as t/ha. PC was determined using near-infrared spectroscopy (Intratec grain analyzer, Foss, Hilleroed, Denmark) and expressed as percentage of GY. NC was computed from GY and PC as $NC = \frac{GY \times PC}{6.25}$ (ISO 16634-2:2016 2016). The TKW was determined as a measure for kernel size [38]. A detailed description of grain-related data can also be found in a previous study focusing on deep root phenotyping [31].

### Root length from minirhizotron imaging

Half of the experimental beds were equipped with minirhizotron tubes, and two root traits were analyzed in this study. Root imaging was done by using a multispectral imaging system, in which a portable trolley system and four multispectral camera systems were used to allow for multivariate image analysis using five wavebands [32]. When taking the image, the portable trolley carried four cameras moved through facility, with a step size of four rows in each movement. Then the side by side cameras were dropped into four adjacent minirhizotron tubes (one camera in each tube) and images of the root were taken along the tube with 5 cm intervals. The root images were made along the upwards facing side of the minirhizotrons, ﻿therefore enable photography of roots covering a soil depth interval of 0.7 m to 2.7 m [6]. The subsequent image analysis delivered an estimate of living root length in each image using the U-Net Neural Network (CNN) architecture to provide automated image segmentation of root structures [47]. A detailed description of the image analysis strategy can be found in a previous study [31]. On average, there were 56 images (4 × 5 cm) used for each minirhizotron tube after editing. The root data were based on root imaging made on 18th June 2018, where 21,057 root images were recorded by four cameras from the 300 minirhizotrons. The imaging of roots was done at late flowering early grain filling since it is widely accepted that the cereal root system reaches the maximum extension after anthesis and limited root development gave been observed during grain filling [48]. Total root length between 1.2 and 2.0 m soil depth (TRL, Additional file 1: Table S3) was expressed as the total length of living roots found in all the images taken from each minirhizotron tube. Root length in four intervals on each tube (IRL, Additional file 1: Table S4) was expressed as the total length of living roots found in all the images taken from each depth interval in each minirhizotron tube. The root data were edited as follows before the genetic analysis:

1. Remove records with failure in getting observation of root in individual image.
2. Divide depth into 8 depth intervals and remove records from above 1.2 m or below 2.0 m soil depth.
3. For each interval, remove records out of mean ± 3sd.
4. remove records from lines without genomic information.

The number of records kept in each step can be found in Additional file 2: Table S5 and the detail depth interval can be found in Additional file 2: Table S6. After editing by rules from step 1 to 4, 14,270 records (images) were kept for further analysis, i.e. to calculate root length from intervals in each tube and root length for each tube.

### Statistical models and methods

Two sets of models, with or without the effects of neighbors from both sides of each row were applied to each trait in this study. These models were designed to analyze the causes of phenotypic variation and decompose the phenotypic variances into different components. The first model (GM1) allocated the phenotypic variances into direct genetic and environmental components, while the second model (GM2) allocated the phenotypic variance into an indirect genetic component of neighbors in addition to the first model. In these two models, the direct genetic components included both genomic and non-genomic effects of the lines own genotype, the indirect genetic components included both genomic and non-genomic effects of the neighbor lines on both sides of the proband, and the environmental components included the non-genetic parts caused by repeated records on each row, the spatial effects over the facility, and residual error.

Spatial effects were included in both models to account differences in measures over the facility due to factors such as soil compaction. As shown in Fig. 1b, modeling of spatial effects was realized by identifying the location for each sample. Then the combination of the location for a sample together with the locations for 5 neighbors from each side of this sample was treated as the spatial effect. The total spatial effects on a row was thus the sum of the joint effects of 11 row locations. When modeling the spatial effects, first the location of one record was treated as the centre point and marked as 0 on the coordinate axis, the 5 locations on the left side were marked as − 1 to − 5, and the 5 locations on the right side were marked as 1 to 5. Then the 11 locations from − 5 to 5 were combined and treated as the spatial effects for the record at the centre point. Border effects at the ends of the beds were handled by including virtual rows outside the facility. This also have the advantage of properly accounting for possible border effects towards the ends of each bed. The effects of neighbors were the effects of the two nearest neighbor lines of each sample, one from the west side and one from the east side.

Specially, for grain-related data, the following models were applied:

GM1 $$\varvec{y} = \varvec{Xb} + \varvec{Z}_{\varvec{l}} \varvec{g} + \varvec{Z}_{\varvec{l}} \varvec{l} + \varvec{Z}_{\varvec{r}} \varvec{r} + \mathop \sum \limits_{{\varvec{i} = 1}}^{11} \varvec{Z}_{{\varvec{s}_{{1\varvec{i}}} }} \varvec{s}_{1i} + \mathop \sum \limits_{{\varvec{i} = 1}}^{11} \varvec{Z}_{{\varvec{s}_{{2\varvec{i}}} }} \varvec{s}_{{2\varvec{i}}} + \varvec{e,}$$

GM2 $$\varvec{y} = \varvec{Xb} + \varvec{Z}_{\varvec{l}} \varvec{g} + \varvec{Z}_{\varvec{l}} \varvec{l} + (\varvec{Z}_{{\varvec{n}_{\varvec{e}} }} + \varvec{Z}_{{\varvec{n}_{\varvec{w}} }} )\varvec{g}_{\varvec{n}} + (\varvec{Z}_{{\varvec{n}_{\varvec{e}} }} + \varvec{Z}_{{\varvec{n}_{\varvec{w}} }} )\varvec{l}_{\varvec{n}} + \varvec{Z}_{\varvec{r}} \varvec{r} + \mathop \sum \limits_{{\varvec{i} = 1}}^{11} \varvec{Z}_{{\varvec{s}_{{1\varvec{i}}} }} \varvec{s}_{{1\varvec{i}}} + \mathop \sum \limits_{{\varvec{i} = 1}}^{11} \varvec{Z}_{{\varvec{s}_{{2\varvec{i}}} }} \varvec{s}_{{2\varvec{i}}} + \varvec{e,}$$

In GM1, ***y*** referred to the vector of phenotypes, which were the records collected from the field or lab, for one trait, the length of ***y*** was equal to the number of records for each trait (1043 for GY; 1044 for TKW; 1045 for PC; 1043 for NC); ***b*** was the vector of fixed effects of interaction between experimental bed and dry/wet harvesting area to correct for the differences might be caused by experimental bed and area, the length of ***b*** was 8 (4 beds × 2 areas); ***g*** was a vector of genomic effects for each line which was the direct genetic effects could be explained by genomic marker information, the length of ***g*** was 84; ***l*** was the vector of line effects for differences between lines not explained by genomic markers, the length of ***l*** was 84; ***r*** was the vector of row effects to account for the repeated records in each row (one record for wet and one record for dry area), the length was 600 (150 rows × 4 beds); $\varvec{s}_{{1\varvec{i}}}$ and $\varvec{s}_{{2\varvec{i}}}$ were the vectors of spatial effects, which accounted for the environmental effects induced by the location of plants, for area 1 (wet) and 2 (dry), respectively, the length of $\varvec{s}_{{1\varvec{i}}}$ and $\varvec{s}_{{2\varvec{i}}}$ were 310 (150 rows × 2 beds + 5 virtual rows on the east boarder + 5 virtual rows on the west boarder); ***X***, ***Z***_***l***_, ***Z***_***r***_, $\varvec{Z}_{{\varvec{s}_{{1\varvec{i}}} }}$, $\varvec{Z}_{{\varvec{s}_{{2\varvec{i}}} }}$ were the corresponding designed matrices of conformable size allocating phenotypic records to ***b***, ***g***, ***l***, ***r***, $\varvec{s}_{{1\varvec{i}}}$, $\varvec{s}_{{2\varvec{i}}}$, and ***e*** was a vector of residual terms with the same length as ***y***, all the incidence matrices had same number of rows which were equal to the length of ***y*** for each trait, and the number of columns for each incidence matrix was same with the length of their corresponding vector of effects. In GM2, in addition to effects in GM1, effects of neighbors were modeled and ***g***_***n***_ was the vector of genomic effects for each neighbor line, which was the indirect genetic effects could be explained by genomic marker information, the length of ***g***_***n***_ was 84; ***l***_***n***_ was the vector of line effects for each neighbor line, which was the indirect genetic effects for differences between lines not explained by genomic markers, the length of ***l***_***n***_ was 84; $\varvec{Z}_{{\varvec{n}_{\varvec{e}} }}$ was the corresponding conformable design matrix allocating phenotypic records to ***g***_***n***_ and ***l***_***n***_ of the neighbor from the east side, $\varvec{Z}_{{\varvec{n}_{\varvec{w}} }}$ was the corresponding conformable design matrix allocating phenotypic records to ***g***_***n***_ and ***l***_***n***_ of the neighbor from the west side. In these models, ***g***, ***l***, ***g***_***n***_, ***l***_***n***_, ***r***, $\varvec{s}_{{1\varvec{i}}}$, $\varvec{s}_{{2\varvec{i}}}$ and ***e*** were random parameters with assumptions $\varvec{g}\sim N\left( {0,\varvec{G}\sigma_{g}^{2} } \right)$, $\varvec{l}\sim N\left( {0,\varvec{I}\sigma_{l}^{2} } \right)$, $\varvec{g}_{\varvec{n}} \sim N\left( {0,\varvec{G}\sigma_{{g_{n} }}^{2} } \right)$, $\varvec{l}_{\varvec{n}} \sim N\left( {0,\varvec{I}\sigma_{{l_{n} }}^{2} } \right)$, $\varvec{r}\sim N\left( {0,\varvec{I}\sigma_{r}^{2} } \right)$, $\varvec{s}_{{1\varvec{i}}} \sim N\left( {0,\varvec{I}\sigma_{{\varvec{s}_{{1\varvec{i}}} }}^{2} } \right)$, $\varvec{s}_{{2\varvec{i}}} \sim N\left( {0,\varvec{I}\sigma_{{\varvec{s}_{{2\varvec{i}}} }}^{2} } \right)$, $\varvec{e}\sim N\left( {0,\varvec{I}\sigma_{e}^{2} } \right)$, and the random effects were assumed to be independent of each other. **G** denoted the genomic additive relationship matrix built following the VanRaden method 1 [20], and **I** denoted an identity matrix.

There were two root traits, TRL and IRL, analyzed in this study. For each minirhizotron tube, there was one TRL record while there were four IRL records since the IRL was recorded for each depth interval along the minirhizotron tube. Therefore, two different models were applied to these two traits.

For TRL data, the following model was applied:

RM1 $$\varvec{y} = \varvec{X}_{1} \varvec{b}_{1} + \varvec{X}_{2} \varvec{b}_{2} + \varvec{Z}_{\varvec{l}} \varvec{g} + \varvec{Z}_{\varvec{l}} \varvec{l} + \mathop \sum \limits_{{\varvec{i} = 1}}^{11} \varvec{Z}_{{\varvec{s}_{\varvec{i}} }} \varvec{s}_{\varvec{i}} + \varvec{e} ,$$

where ***b***_***1***_ was the vector of fixed effects of experimental bed to correct for differences that might be caused by bed, the length of ***b***_***1***_ was 2 because only two beds had minirhizotrons; ***b***_***2***_ was the vector of fixed effects of camera to correct for the difference caused by imaging camera, the length of ***b***_***2***_ was 4 (4 cameras); ***s*** was the vector of spatial effects accounted for the environmental effects induced by the location of plants, the length of ***s*** was 310 (150 rows × 2 beds + 5 virtual rows on the east boarder + 5 virtual rows on the west boarder); ***X***_***1***_, ***X***_***2***_, $\varvec{Z}_{{\varvec{s}_{\varvec{i}} }}$ were the corresponding conformable design matrixes allocating phenotypic records to ***b***_***1***_, ***b***_***2***_ and ***s***. In these models, ***s***_***i***_ was random parameter with $\varvec{s}_{\varvec{i}} \sim N\left( {0,\varvec{I}\sigma_{{s_{i} }}^{2} } \right)$ and was assumed independent from other effects. ***y***, ***g***, ***l***, ***e***, ***Z***_***l***_, **G** and **I** were same as in GM1.

In addition, for IRL data, the following model for repeated records was applied:

RM2 $$\varvec{y} = \varvec{X}_{1} \varvec{b}_{1} + \varvec{X}_{2} \varvec{b}_{2} + \varvec{X}_{3} \varvec{b}_{3} + \varvec{Z}_{\varvec{l}} \varvec{g} + \varvec{Z}_{\varvec{l}} \varvec{l} + \varvec{Z}_{\varvec{r}} \varvec{r} + \mathop \sum \limits_{{\varvec{i} = 1}}^{11} \varvec{Z}_{{\varvec{s}_{\varvec{i}} }} \varvec{s}_{\varvec{i}} + \varvec{e} ,$$

where ***b***_***3***_ was the vector of fixed effects of depth interval to correct for the difference might be caused by soil depth, the length of ***b***_***3***_ was 4 (4 intervals); ***X***_***3***_ was the corresponding designed matrix allocating phenotypic records to ***b***_***3***_, ***X***_***3***_ had same number of rows which were equal to the length of ***y***, and the number of columns was same with the length of ***b***_***3***_. ***y***, ***g***, ***l***, ***r***, ***e***, ***Z***_***l***_, ***Z***_***r***_, **G** and **I** were same as in GM1. ***b***_***1***_, ***b***_***2***_, ***s***_***i***_, ***X***_***1***_, ***X***_***2***_ and $\varvec{Z}_{{\varvec{s}_{\varvec{i}} }}$ were same as in RM1.

Compared with RM1 for TRL, RM2 for IRL included ***b***_***3***_ to account and correct for the effect of depth interval along each minirhizotron tube. Besides, because of repeated records for IRL compared with TRL, RM2 also included ***r*** to account for the random row effects due to the repeated records on each row.

The models applied in this study were developed based on preliminary investigations. First, genomic by water gradient effects were investigated and the variance of genomic by water gradient effects were all not significantly different from 0. Secondly, spatial effects were investigated in each wet or dry area separately, and the results showed that the variances of spatial effects were different in the two divisions of the water gradient. Thirdly, neighbor effects were first modeled as different effects of varieties east or west of the proband. Initial results showed that the correlation of neighbor effects from two sides was close to 1 and therefore could be combined into one effect with common variance. Finally, the neighbor effects were also modeled for root traits but it was difficult to carry out prediction using such complex model on these traits due to the few rows with minirhizotron tubes. Therefore, the simplified models described above were applied and compared in this study.

### Estimation of variance components

All models were analyzed by the restricted maximum likelihood method (REML) using the DMU software package [49]. Relative variance component (RVC), which was the percentage of each weighted VC accounted for the total phenotypic variance for each line, was computed. The phenotypic variance of line means was calculated as the sum of weighted variance components:

$$\sigma_{{P_{iBM1} }}^{2} = \overline{G} \sigma_{g}^{2} + \sigma_{l}^{2} + \sigma_{r}^{2} /n_{r} + 11\sigma_{{s_{i} }}^{2} /n_{{s_{i} }} + \sigma_{e}^{2} /n_{e} ,$$

$$\sigma_{{P_{iBM2} }}^{2} = \overline{G} \sigma_{g}^{2} + \sigma_{l}^{2} + 2\overline{G} \sigma_{{g_{n} }}^{2} + 2\sigma_{{l_{n} }}^{2} + \sigma_{r}^{2} /n_{r} + 11\sigma_{{s_{i} }}^{2} /n_{{s_{i} }} + \sigma_{e}^{2} /n_{e} ,$$

$$\sigma_{{P_{RM1} }}^{2} = \overline{G} \sigma_{g}^{2} + \sigma_{l}^{2} + 11\sigma_{s}^{2} /n_{s} + \sigma_{e}^{2} /n_{e} ,$$

$$\sigma_{{P_{RM2} }}^{2} = \overline{G} \sigma_{g}^{2} + \sigma_{l}^{2} + \sigma_{r}^{2} /n_{r} + 11\sigma_{s}^{2} /n_{s} + \sigma_{e}^{2} /n_{e} ,$$

where $\overline{G}$ was the average diagonal of the **G** matrix, *n*_*r*_ was the average number of rows for each line, $n_{{s_{i} }}$ and *n*_*s*_ were the average numbers of replicates for each line, and *i* was 1 (wet) or 2 (dry), and *n*_*e*_ was average number of replicates across all fields for each line. For the grain-related traits which having two areas (wet and dry), the phenotypic variance was calculated for each area separately due to the difference in spatial variance in the two area levels. The RVC of ***g*** was equivalent to estimated narrow sense heritability ($\widehat{{h^{2} }}$) in this study, which was the narrow sense heritability (*h*^*2*^) of a line mean and thus was a function of the experimental design (number of replicates etc.) as is commonly done in the plant breeding community.

In addition, the coefficient of genomic variation (***c***_***v***_) was calculated for each trait, according to: $c_{v} = \frac{{\sigma_{g} }}{{\overline{x} }}$, in which $\sigma_{g}$ was the square root of genomic variance and $\overline{x}$ was the average of observations for each trait.

### Genomic prediction and cross-validation

To estimate the accuracy of genomic breeding values (GEBVs), a leave-one-line-out cross-validation (LOO-CV) strategy was applied. In each LOO-CV round, the phenotypes of one line were masked and then all other lines were used to train the prediction model and to predict the line with phenotypes masked and this was continued until all lines were predicted.

Before LOO-CV, the whole dataset was used to estimate VCs and fixed effects. Corrected phenotypes (*y*_*c*_) were computed by subtracting the estimates of the fixed effects. Accuracy of prediction (ACC) was measured as correlation between average *y*_*c*_ and GEBVs, and then scaled by square root of $\widehat{{h^{2} }}$ ($\sqrt {cor\left( {\overline{y}_{c} ,\widehat{g}} \right)^{2} /\widehat{{h^{2} }}) }$) [42], where $\overline{y}_{c}$ was the average *y*_*c*_ for each line. This scaling yields an estimate of the correlation between GEBV and underlying true breeding values for lines that only have genomic information but no own phenotypic record. Furthermore, to assess inflation of GEBVs, the regression coefficient of $\overline{y}_{c}$ on $\widehat{g}$ was calculated, where a regression of unity indicates no inflation.

## Supplementary information

**Additional file 1: Table S1.** Genotypic data**. Table S2.** Phenotypic data for grain-related traits**. Table S3.** Phenotypic data for total root length between 1.2 and 2 m depth**. Table S4.** Phenotypic data for root length in four intervals on each tube.

**Additional file 2: Table S5.** Editing rules for root data and number of records kept in each step**. Table S6.** Depth interval, range of tube (mm) and soil (cm) depth together with number of records.

## Data Availability

All data generated or analyzed during this study are included in this published article and its supplementary information files.

## Abbreviations

ACC: Accuracy of prediction of genomic breeding values
CNN: Convolutional Neural Network
G: Genomic relationship matrix
GBLUP: Genomic best linear unbiased prediction
GEBVs: Genomic expected breeding values
GP: Genomic prediction
GS: Genomic selection
GWAS: Genome-wide association study
GY: Grain yield
IGE: Indirect genetic effect
IRL: Root length in four intervals on each tube
LOO-CV: Leave-one-line-out cross-validation
LSO-CV: Leave-set-out cross-validation
MAS: Marker-assisted selection
NC: Total nitrogen content
PA: Predictive ability
PC: Protein concentration
REML: Restricted maximum likelihood
RVC: Relative variance component
TKW: Thousand-kernel weight
TRL: Total root length between 1.2 and 2 m depth
VC: Variance component
