# Supplementary material for: Genomic prediction of yield and root development in wheat under changing water availability
Source: Plant Methods. 2020 Jul 1;16:90. doi: 10.1186/s13007-020-00634-0 (PMC7329460; doi:10.1186/s13007-020-00634-0)
Supplement: Supplementary file 2 — Additional file 2: Table S5. Editing rules for root data and number of records kept in each step. Table S6. Depth interval, range of tube (mm) and soil (cm) depth together with number of records. [file 13007_2020_634_MOESM2_ESM.docx]

# Genomic prediction of yield and root development in wheat under changing water availability

Xiangyu Guo^1*^, Simon F. Svane^2^, Winnie S. Füchtbauer^3^, Jeppe R. Andersen^4^, Just Jensen^1^, Kristian Thorup-Kristensen^2^

^1^Center for Quantitative Genetics and Genomics, Aarhus University, 8830 Tjele, Denmark

^2^Department of Plant and Environmental Science, University of Copenhagen, 1871 Frederiksberg, Denmark

^3^Sejet Plant Breeding I/S, 8700 Horsens, Denmark

^4^Nordic Seed A/S, 8300 Odder, Denmark

***Corresponding Author**

Xiangyu Guo

[xiangyu.guo@mbg.au.dk](mailto:xiangyu.guo@mbg.au.dk)

# Tables

**Table S5** Editing rules for root data and number of records kept in each step

| Step | Rule | No. of records |
| --- | --- | --- |
| 0 | full data | 21,057 |
| 1 | remove records with failure in getting observation of root in individual image | 20,479 |
| 2 | divide depth into 8 intervals and remove records with soil depth larger than 2 m (4 m along tube) or smaller than 1.2 m (2 m along tube) | 16,211 |
| 3 | for each interval, remove records out of mean±3sd | 15,960 |
| 4 | remove records from lines without genomic information | 14,270 |

Soil depth = (Tube depth-420mm) × sin(23.5^o^) + 570mm

Table S6 Depth interval, range of tube (mm) and soil (cm) depth together with number of records

| Interval | Min tube depth | Max tube depth | Min soil depth | Max soil depth | No. records |
| --- | --- | --- | --- | --- | --- |
| 1 | 0 | 1,500 | 0 | 100.06 | 0 |
| 2 | 1,500 | 2,000 | 100.06 | 120.00 | 0 |
| 3 | 2,000 | 2,500 | 120.00 | 139.94 | 3,529 |
| 4 | 2,500 | 3,000 | 139.94 | 159.88 | 3,753 |
| 5 | 3,000 | 3,500 | 159.88 | 179.81 | 3,508 |
| 6 | 3,500 | 4,000 | 179.8 | 199.75 | 3,480 |
| 7 | 4,000 | 4,500 | 199.75 | 219.69 | 0 |
| 8 | 4,500 | 5,006 | 219.69 | 239.87 | 0 |

Soil depth = (Tube depth-420mm) × sin(23.5^o^) + 570mm
